# Prediction of postoperative inflammatory complications after esophageal cancer surgery based on early changes in the C-reactive protein level in patients who received perioperative steroid therapy and enhanced recovery after surgery care: a retrospective analysis

**DOI:** 10.1186/s12885-017-3831-2

**Published:** 2017-12-04

**Authors:** Kazuki Kano, Toru Aoyama, Tetsushi Nakajima, Yukio Maezawa, Tsutomu Hayashi, Takanobu Yamada, Tsutomu Sato, Takashi Oshima, Yasushi Rino, Munetaka Masuda, Haruhiko Cho, Takaki Yoshikawa, Takashi Ogata

**Affiliations:** 1 Department of Gastrointestinal Surgery, Kanagawa Cancer Center, 2-3-2, Nakao, Asahi-ku, Yokohama, Kanagawa 241-8515 Japan; 2 Department of Surgery, Yokohama City University, 3-9, Fukuura, Kanazawa-ku, Yokohama, Kanagawa 236-0004 Japan

**Keywords:** Esophageal cancer, Complication, Steroid therapy, C-reactive protein, Enhanced recovery after surgery care, Predictor

## Abstract

**Background:**

Serum C-reactive protein (CRP) level can be an indicator of the early stage of infectious complications. However, its utility in advanced esophageal cancer patients who receive radical esophagectomy with two- or three-field lymph node dissection with perioperative steroid therapy and enhanced recovery after surgery (ERAS) care is unclear.

**Methods:**

The present study retrospectively examined 117 consecutive esophageal cancer patients who received neoadjuvant chemotherapy followed by radical esophagectomy. All patients received perioperative steroid therapy and ERAS care. The utility of the CRP value in the early detection of serious infectious complications (SICs) was evaluated based on the area under the receiver operating characteristic curve (AUC). Univariate and multivariate logistic regression analyses were performed to identify the risk factors for SICs.

**Results:**

SICs were observed in 20 patients (17.1%). The CRP level on postoperative day (POD) 4 had superior diagnostic accuracy for SICs (AUC 0.778). The cut-off value for CRP was determined to be 4.0 mg/dl. A multivariate analysis identified CRP ≥ 4.0 mg/dl on POD 4 (odds ratio, 18.600; 95% confidence interval [CI], 4.610–75.200) and three-field lymph node dissection (odds ratio, 7.950; 95% CI, 1.900–33.400) as independent predictive factors.

**Conclusions:**

CRP value on POD 4 may be useful for predicting SICs in esophageal cancer patients who receive radical esophagectomy with perioperative steroid therapy and ERAS care. This result may encourage the performance of imaging studies to detect the focus and thereby lead to the early medical and/or surgical intervention to improve short-term outcomes.

## Background

Preoperative chemo(radio)therapy and surgery have been established as the standard treatment for locally advanced esophageal cancer [1, 2]. Although recent advances in esophagectomy have decreased mortality, the morbidity remains high at 30%-65% [3, 4]. Among surgical morbidities, infectious complications (ICs) can be lethal if the initiation of effective treatment is delayed. However, the early clinical features of ICs are nonspecific and difficult to distinguish from normal postoperative inflammatory responses associated with surgical invasion [5]. Therefore, ICs are often diagnosed after patients develop apparent clinical symptoms. Indeed, the median time to the diagnosis ICs was reportedly up to 12 days after surgery [6]. To improve the short-term outcomes, approaches other than symptom observation must be adopted for the early detection of ICs.

Several studies have reported the utility of serum C-reactive protein (CRP) in predicting ICs before clinical signs and symptoms develop [7, 8]. However, previous studies have included patients with esophagogastric junctional adenocarcinoma, and some were performed in Western populations. The Eastern surgical procedure, which was defined as radical esophagectomy with extended lymph node dissection, the cervical and upper mediastinal as well as middle-lower mediastinal and abdominal lymph node dissection [9, 10], for esophageal squamous cell carcinoma located in the thoracic esophagus is a highly invasive surgery, that is completely different from the Ivor-Lewis procedure for esophageal adenocarcinoma located in the distal esophagus [11]. Furthermore, perioperative managements, such as steroid therapy and enhanced recovery after surgery (ERAS) care, that have been introduced in many hospitals to reduce the morbidity and mortality, were recently reported to reduce the postoperative serum CRP levels [12–15], making ICs more difficult to diagnose in the early period. Thus, the findings from previous reports on the utility of CRP levels in the early prediction of ICs cannot be generalized.

The aim of this study was to assess whether early changes in the serum CRP can be used to predict ICs in advanced esophageal cancer patients who received esophagectomy and two- or three-field lymph node dissection with perioperative steroid therapy and ERAS care.

## Methods

### Patient data

The patients were selected from the medical records of consecutive patients who underwent esophagectomy for esophageal cancer at Kanagawa Cancer Center from January 2011 to September 2015. The patients met the following inclusion criteria: (1) histologically proven primary esophageal squamous cell carcinoma located at thoracic esophagus, (2) clinical stage I to III (excluding T4) disease as evaluated using the 7th edition of the tumor-node-metastasis classification established by the Union for International Cancer Control [16], and (3) neoadjuvant chemotherapy followed by curative resection with radical lymph node dissection.

### Preoperative chemotherapy

The patients received two courses of cisplatin plus 5-fluorouracil. Cisplatin was administered at a dose of 80 mg/m^2^ by intravenous drip infusion on day 1, and 5-fluorouracil was administered at a dose of 800 mg/m^2^ by continuous infusion on days 1-5 [1].

### Surgical procedure

Surgical resection was generally performed 4-6 weeks after the completion of chemotherapy. Our standard procedures consisted of open subtotal esophagectomy via right anterolateral thoracotomy, reconstruction with a gastric tube through the posterior mediastinal route or retrosternal route, and anastomosis in the cervical incision. In principle, two-field lymph node dissection is indicated when tumors are located at the middle thoracic to lower thoracic esophagus, while three-field is applied for upper thoracic tumors. Multiple drains were placed; one to the posterior side of the thoracic cavity and the others on either side of the neck. A feeding tube was routinely placed at the stomach or duodenum.

### Perioperative care

All of the patients received perioperative management by the clinical path based on the ERAS program, which routinely included antibiotic prophylaxis and steroid therapy. Cefazolin (1 g) was administered 30 min before surgical incision and then again every 3 hours during surgery and at 2 g on postoperative day (POD) 1. Methylprednisolone was administered at a dose of 500 mg on the day of surgery, 250 mg on POD 1, and 125 mg on POD 2 [13, 14]. Our ERAS program satisfied the 15 items proposed by Fearon et al. [17]. Briefly, the patients were allowed to eat 30% rice porridge until midnight the day before the surgery and were required to drink the contents of two 500-ml plastic bottles containing oral rehydration solution by 3 h before surgery. Intraoperatively, we conducted epidural anesthesia with morphine for pain control during surgery. Previous study showed the use of the epidural anesthesia with morphine has clinical benefits such as, a selective analgesia with no motor or sympathetic blockade and a long analgesia at low use of rescue medication [18, 19]. However, the use of the epidural anesthesia with morphine could cause delayed respiratory depression and apnea as late as 12 hours after administration [20]. Therefore, the patients remained on ventilation for 12 hours after surgery. After 12 hours, we carefully observe respiratory condition and extubate. Ambulation and enteral nutrition was started on POD 1. Oral intake was initiated on POD 6, beginning with water and gelatinous foods. The patients began to eat solid food on POD 9, starting with rice gruel and soft food and progressing in three steps to regular food intake.

### Definition of surgical complications and measurement of CRP

All data were retrospectively retrieved from the patients’ records. ICs were defined as complications of anastomotic leakage, pneumonia, abdominal abscess, and/or pyothorax according to the Clavien-Dindo classification [21] occurring during hospitalization within 30 days after surgery. Of these, ICs ≥ grade IIIa were defined as serious ICs (SICs). The complications were assessed based on the clinical symptoms, blood tests, and X-ray imaging at POD 1, 2, 4, 6, 8, and thereafter. If ICs were suspected, precise examinations, such as computed tomography, esophagography, and esophagoduodenoscopy, were performed.

### Statistical analyses

A two-sided *P* value < 0.05 was considered significant. Continuous data are presented as the median with the range. The Mann-Whitney U test and Fisher's exact test were employed to evaluate the differences in continuous and categorical variables, respectively. The patients were classified as those with SICs (SICs group) and those without SICs (NSICs group). The diagnostic accuracy was determined based on the area under the receiver operating characteristic (ROC) curve (AUC) [22]. The optimal cut-off value of CRP was determined by maximizing Youden’s index. The optimum value of CRP was then determined based on the AUC and the earliest prediction of SICs. The predictive value of CRP, categorized as high or low by the cut-off value at the optimum point, was examined using univariate and multivariate logistic regression analyses. All statistical analyses were performed with EZR (Saitama Medical Center, Jichi Medical University, Saitama, Japan), which is a graphical user interface for R (The R Foundation for Statistical Computing, Vienna, Australia). More precisely, it is a modified version of R commander designed to add statistical functions frequently used in biostatistics [23].

## Results

### Patient characteristics

A total of 208 patients underwent esophagectomy for esophageal squamous cell carcinoma between January 2011 and September 2015. Excluding 3 patients with no survival information available, 7 patients who were not diagnosed with squamous cell carcinoma, 74 patients who did not receive neoadjuvant chemotherapy, and 7 patients who did not receive curative resection, one hundred and seventeen of these patients were eligible for the present study (56.3%). The patient characteristics are summarized in Table 1. The SICs group received three-field lymph node dissection more frequently (*p* = 0.023) and had greater blood loss (*p* = 0.018) than the NSICs group.

**Table 1:** A comparison of patients’ characteristics and surgical findings between the patients with and without postoperative serious infectious complications

Variables	All Patients (*n* = 117)	SICs group (*n* = 20)	NSICs group (*n* = 97)	*p* value
Age (years), median (range)	66 (48-77)	68 (50-77)	66 (48-77)	0.289
Gender				0.356
Male	94 (80.3%)	18 (90.0%)	76 (78.4%)	
Female	23 (19.7%)	2 (10.0%)	21 (21.6%)	
Preoperative body mass index (kg/m2), median (range)	21.1 (15.4-28.9)	21.8 (17.8-26.7)	20.5 (15.4-28.9)	0.205
Preoperative serum albumin (g/dl), median (range)	4.1 (2.3-6.4)	4.1 (3.2-4.4)	4.1 (2.3-6.4)	0.202
ASA-PS				0.779
1	17 (14.5%)	2 (10.0%)	15 (15.5%)	
2	99 (84.6%)	18 (90.0%)	81 (83.5%)	
3	1 (0.9%)	0 (0.0%)	1 (1.0%)	
Main tumor location				0.091
Upper thoracic esophagus	16 (13.7%)	6 (30.0%)	10 (10.3%)	
Middle thoracic esophagus	61 (52.1%)	9 (45.0%)	52 (53.6%)	
Lower thoracic esophagus	40 (34.2%)	5 (25.0%)	35 (36.1%)	
UICC clinical T factor before neoadjuvant chemotherapy				0.235
cT1	2 (1.7%)	1 (5.0%)	1 (1.0%)	
cT2	39 (33.3%)	8 (40.0%)	31 (32.0%)	
cT3	76 (65.0%)	11 (55.0%)	65 (67.0%)	
UICC clinical N factor before neoadjuvant chemotherapy				0.841
cN0	50 (42.7%)	8 (40.0%)	42 (43.3%)	
cN1	66 (56.4%)	12 (60.0%)	54 (55.7%)	
cN2	1 (0.9%)	0 (0.0%)	1 (1.0%)	
UICC clinical stage before neoadjuvant chemotherapy				0.090
IB	23 (19.7%)	7 (35.0%)	16 (16.5%)	
IIA	27 (23.1%)	1 (5.0%)	26 (26.8%)	
IIB	18 (15.4%)	2 (10.0%)	16 (16.5%)	
IIIA	48 (41.0%)	10 (50.0%)	38 (39.2%)	
IIIB	1 (0.9%)	0 (0.0%)	1 (1.0%)	
Lymph node dissection				0.023
Two-field	95 (81.2%)	12 (60.0%)	83 (85.6%)	
Three-field	22 (18.8%)	8 (40.0%)	14 (14.4%)	
Operation time (min), median (range)	400 (298-593)	430.0 (345-593)	395 (298-593)	0.111
Intraoperative blood loss (ml), median (range)	420 (110-3000)	682.5 (185-3000)	400 (110-2350)	0.018

*SICs* Serious infectious complications, *ASA-PS* American Society of Anesthesiologists Physical Status, *UICC* Union for International Cancer Control

### Surgical morbidity and mortality

SICs were observed in 20 patients (17.1%). The details of the complications and duration from surgery to their diagnosis are shown in Table 2. The median duration until the diagnosis of any SICs was 7 days (range: 4-14).

**Table 2:** Details of serious infectious complications and duration from surgery to the diagnosis of those complications

	Grade according to Clavein-Dindo classification					Total (%)	Duration to diagnose SICs, median (range)
Complications	3a	3b	4a	4b	5		
Anastomotic leakage	16	0	0	0	0	16 (13.7%)	6 (4-10)
Abdominal abscess	0	1	0	0	0	1 (0.9%)	7 (7)
Pneumonia	1	0	1	0	0	2 (1.7%)	6 (5-7)
Pyothorax	5	0	0	0	0	5 (4.3%)	10 (6-14)
Total	22	1	1	0	0	24 (20.5%)	7 (4-14)

There is some overlapping.

*SICs* Serious infectious complications, *POD* Postoperative day

### Postoperative CRP level with SICs

**Fig. 1:**
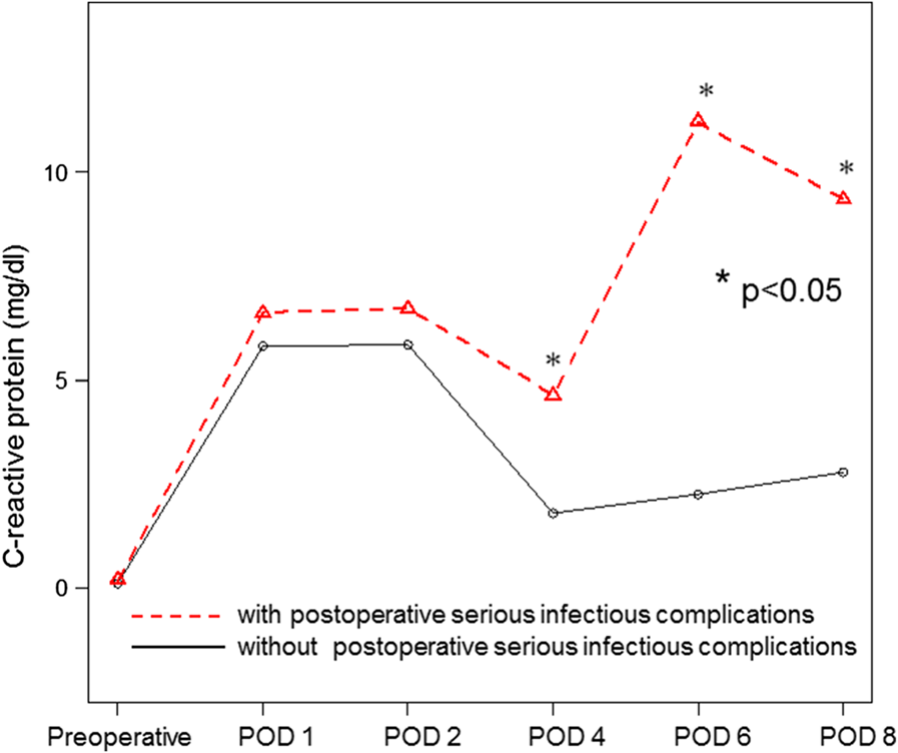
Changes in the C-reactive protein (CRP) levels between patients with and without serious infectious complications (SICs). The CRP levels were significantly different on postoperative days 4, 6, and 8. The optimum CRP value for the prediction of SICs was determined to be that measured on POD 4

**Fig. 2:**
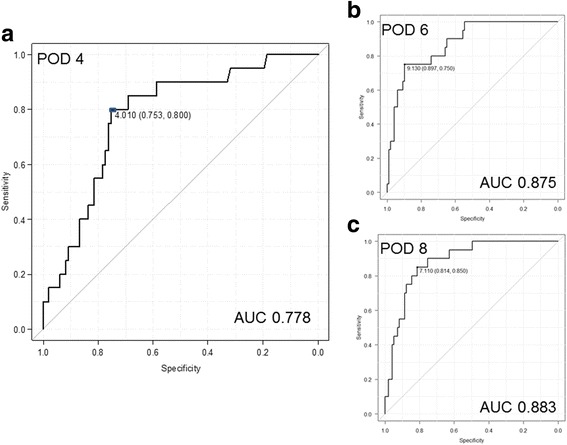
Diagnostic accuracy was determined based on the area under the receiver operating characteristic (ROC) curve (AUC) (CRP on POD 4, 6, and 8) for predicting SICs. The AUC for prediction by CRP was 0.778 (95% CI, 0.673-0.884) on POD 4 (**a**), 0.875 (95% CI, 0.799-0.952) on POD 6 (**b**), and 0.883 (95% CI, 0.813-0.953) on POD 8 (**c**)

The changes in the CRP level after esophagectomy are shown in Fig. 1. The preoperative CRP level was not markedly different between the SICs and NSICs groups. After surgery, the CRP level reached its first peak on POD 1 and 2, with no significant differences between the two groups, and then decreased to its lowest value on POD 4. However, the subsequent CRP levels on POD 4, 6, and 8 were significantly higher in the SICs group than in the NSICs group. The AUC for prediction by CRP was 0.778 (95% CI, 0.673-0.884) on POD 4 (Fig. 2a), 0.875 (95% CI, 0.799-0.952) on POD 6 (Fig. 2b), and 0.883 (95% CI, 0.813-0.953) on POD 8 (Fig. 2c). Considering the AUC and earliest prediction of SICs, the optimum cut-off value of CRP was determined to be 4.0 mg/dl on POD 4. By this cut-off, 40 patients had high CRP with median of 6.95 mg/dl (range: 4.01-28.51), while 77 patients had CRP with median of 1.50 mg/dl (range: 0.13-3.99). Among 40 patients with high CRP levels, 16 developed SICs; anastomotic leakage in 12 patients, pneumonia in 2, abdominal abscess in 1, and pyothorax in 3. The sensitivity and specificity were 80.0% and 75.3%, respectively, and the negative and positive predictive values (NPV and PPV) were 94.8% and 40.1%, respectively.

### Risk factors for SICs

Table 3 shows the results of univariate and multivariate analyses (Table 3 **)**. Among these, CRP ≥ 4.0 mg/dl on POD 4 (odds ratio, 18.600; 95% CI, 4.610–75.200) and three-field lymph node dissection (odds ratio, 7.950; 95% CI, 1.900–33.400) were identified as significant independent predictive factors for SICs.

**Table 3:** Predictive factors for serious infectious complications

Factors	Number of patients (%)	Univariate			Multivariate		
		OR	95% CI	*p* value	OR	95% CI	*p* value
Age (years)				0.271			
≤66	60 (51.3%)	1.000					
>66	57 (48.7%)	1.730	0.651-4.620				
Gender				0.246			
Female	23 (19.7%)	1.000					
Male	94 (80.3%)	2.490	0.534-11.600				
Preoperative body mass index (kg/m2)				0.183			
≤21	57 (48.7%)	1.000					
>21	60 (51.3%)	1.980	0.726-5.380				
ASA-PS				0.531			
1	17 (14.5%)	1.000					
2/3	100 (85.5%)	1.650	0.346-7.840				
Preoperative serum albumin (g/dl)				0.900			
≥4.1	60 (51.3%)	1.000					
<4.1	57 (48.7%)	1.060	0.406-2.790				
UICC clinical T factor before neoadjuvant chemotherapy				0.308			
cT1-2	41 (35.0%)	1.000					
cT3	76 (65.0%)	0.602	0.226-1.600				
UICC clinical N factor before neoadjuvant chemotherapy				0.786			
cN0	50 (42.7%)	1.000					
cN1-2	67 (57.3%)	1.150	0.430-3.050				
Lymph node dissection				0.011			0.005
Two-field	95 (81.2%)	1.000			1.000		
Three-field	22 (18.8%)	3.950	1.370-11.400		7.950	1.900-33.400	
Operation time (min)				0.135			
≤400	59 (50.4%)	1.000					
>400	58 (49.6%)	2.150	0.788-5.840				
Intraoperative blood loss (ml)				0.237			
≤420	61 (52.1%)	1.000					
>420	56 (47.9%)	1.810	0.678-4.810				
CRP on POD4 (mg/dl)				<0.001			<0.001
≤4.0	77 (65.8%)	1.000			1.000		
>4.0	40 (34.5%)	12.200	3.710-39.900		18.600	4.610-75.200	

*SICs* Serious infectious complications, *CI* Confidence interval, *OR* odds ratio, *ASA-PS* American Society of Anesthesiologists Physical Status, *CRP* C-reactive protein; *POD*, postoperative day, *UICC* Union for International Cancer Control

## Discussion

The present study examined whether CRP levels can predict SICs in 117 advanced esophageal squamous cell carcinoma patients who received neoadjuvant chemotherapy followed by curative resection with perioperative steroid therapy and ERAS care. This study found that a CRP level exceeding 4.0 mg/dl on POD 4 was useful for predicting SICs in esophageal squamous cell carcinoma patients who received radical esophagectomy with perioperative steroid therapy and ERAS care. A high CRP level on POD 4 may encourage the performance of imaging studies to detect the focus and thereby lead to early medical and/or surgical intervention.

The cut-off CRP value was 4.0 mg/dl on POD 4 in the present study. Compared with previous studies examining the utility of CRP in predicting SICs, our surgical approach was highly invasive, but the operation time and blood loss were similar [24, 25]. However, the cut-off CRP value was much lower than in previous studies, ranging from 11.1 to 18.0 mg/dl on POD 3 or 4 [7, 8, 26]. This low cut-off CRP value may be explained by the use of steroid therapy and ERAS in our study, which helped reduce the surgical stress-induced inflammatory responses [12–15]. Several studies reported that the postoperative CRP levels were decreased to nearly half in patients who underwent esophagectomy and received perioperative steroid therapy [13, 14]. Furthermore, Chen et al. found that the postoperative CRP levels on POD 1, 3, and 7 were significantly lower in patients who received perioperative care with fast track surgery than in others [15].

Although the cut-off CRP value in the present study was low, the sensitivity and specificity were around 70%-80%, which was concordant with the values in previous studies [7, 8, 26, 27]. Furthermore, the high NPV of 94.8% in the present study suggested that SICs can be ruled out when the CRP is less than 4.0 mg/dl on POD 4 [28]. However, the PPV of 40.1% might be too low to support the accurate diagnosis of SICs based on CRP values. Therefore, patients with CRP levels ≥ 4.0 mg/dl on POD 4 must be screened for SICs by further diagnostic measures, like X-rays, upper gastrointestinal series, or computed tomography. CRP measurement on POD 4 is nonspecific, but it is nevertheless helpful since it encourages the performance of further studies to detect the focus [27].

The earliest point for the successful prediction by the CRP level was POD 4 in the present study, which has clinical impact as physicians can initiate early goal-directed therapy, thereby improving patients’ short-term outcome [5, 24]. Generally, the half-life of CRP is 19 h [29]. Several investigators have reported that the CRP level peaked on POD 2 before normalizing on POD 3 following various types of surgery [7, 28, 29]. Because this study did not measure the CRP level on POD 3, it remains unclear whether SICs could be predicted on POD 3. However, the CRP level on POD 6 and 8 had high diagnostic accuracy in the present study, possibly suggesting that CRP increased with the progression of SICs; however, no effective treatment was introduced, possibly due to the lack of any clinical sign of SICs. In other words, SICs may actually start on POD 4 rather than simply being detected on that day. This hypothesis is supported by the findings from previous studies [27, 30], as Deitmar et al. showed that elevated CRP levels precede the development of SICs by 3 days [30].

Our results demonstrated that three-field lymph node dissection had more complications than two-field. According to the previous reports, it is controversial whether the addition of lateral neck dissection may lead to SICs [9]. Recent meta-analysis showed that three-field lymph node dissection had more complications than two-field [31]. In this study, three-field lymph node dissection was applied for upper thoracic tumors, which might have resulted in SICs. Although the difference between two- or three-field lymph node dissection is just whether lateral neck dissection is added or not, technical difficulties in surgery for the proximal esophagus might increase SICs [32]. In fact, postoperative complications had been reported in as high as 61.5 to 71.4% of patients with the upper thoracic esophageal cancer [33].

The present study is associated with several potential limitations. First, it was a retrospective single-center study with a small sample size. Second, there is no standard type, period, or dose of perioperative steroid therapy. The perioperative ERAS program also differs by hospital. Thus, the cut-off CRP value likely differs depending on the perioperative management regimen adopted by a given hospital. Third, the present study only investigated the outcomes following open subtotal esophagectomy via right anterolateral thoracotomy. Recently, minimally invasive surgery has been introduced [34]. Because the invasiveness of surgery is different, the cut-off CRP value may also be different with minimally invasive surgery. To confirm the present results, prospective study is necessary.

## Conclusions

A high CRP level ≥ 4.0 mg/dl on POD 4 may predict SICs in esophageal cancer patients who received neoadjuvant chemotherapy followed by curative resection with perioperative steroid therapy and ERAS care. This result may encourage the performance of imaging studies to detect the focus and thus lead to early medical and/or surgical intervention, thereby helping to improve the short-term outcome.

## Abbreviations

AUC: Area under the receiver operating characteristic curve
CI: Confidence interval
CRP: C-reactive protein
ERAS: Enhanced recovery after surgery
IRB: Institutional Review Board
NPV: Negative predictive value
POD: Postoperative day
PPV: Positive predictive value
ROC: Receiver operating characteristic
SICs: Serious infectious complications
